# Study of Effect of Coil Movement on Growth Conditions of SiC Crystal

**DOI:** 10.3390/ma16010281

**Published:** 2022-12-28

**Authors:** Shengtao Zhang, Hao Fu, Tie Li, Guofeng Fan, Lili Zhao

**Affiliations:** 1 School of Chemistry and Chemical Engineering, Harbin Institute of Technology, Harbin 150001, China; 2 Harbin KY Semiconductor Inc., Harbin 150028, China; 3 Soft-Impact China (Harbin) Ltd., Harbin 150028, China

**Keywords:** silicon carbide, coil movement, growth interface, temperature gradient

## Abstract

SiC substrates have outstanding advantages over traditional materials in power device application, and are mainly prepared by a physical vapor transport method (PVT). Whether the PVT furnace works by resistance heating or induction heating, both face the problem of the deterioration of growth conditions during a long-term process. The relative position of the thermal field directly affects the crystal growth conditions, but the law of specific influence and the change in physical environment inside the thermal field have not been made sufficiently clear and lack systematic research. Therefore, SiC single crystal growth, with different directions and rates in the direction of movement of the heating module, was modeled using a simulation method, and the law of variation of the physical field, including heat flux, temperature, powder porosity and growth rate parameters under different schemes, was analyzed. The study indicates that the decay of raw materials is the primary reason why growth conditions cannot be maintained. The results verified that different coils’ modes of movement have different effects on the improvement or adjustment of SiC crystals’ growth conditions. Under the same temperature control conditions, the coils’ movement rates of 200 μm/h, 0, −200 μm/h and −400 μm/h correspond to the average growth rates of 140, 152, 165 and 172 μm/h, respectively. The results show that downward displacement of the coils is beneficial in compensating for the deterioration of growth conditions, but it is easier to form convex surfaces and is not conducive to expanding diameter growth. This also verifies that the desired crystal growth state can be obtained by adjusting the position of the thermal field.

## 1. Introduction

Silicon carbide (SiC) exhibits unique thermal and mechanical properties and is thus suitable for a wide range of technological applications in different solid-state forms, including powder, ceramics and single crystal [1,2,3,4]. Among these, 4H-SiC single crystal is the most important, as power devices based on SiC substrate have outstanding advantages of high power, high frequency and high temperature application fields [5,6]. Both a solution method and a gas phase method can be used to prepare SiC crystals, but the PVT method is the most mature process at present [7,8,9]. Its basic principle is to place the source material and seed crystal in a high-temperature zone and a low-temperature zone, respectively, and the vapor components are transported to the seed crystal surface for deposition, which makes the crystal grow to a certain thickness [10]. The difference in equipment is that between induction heating and resistance heating. Subsequently, the grown crystals undergo a series of processes to obtain epitaxial substrate materials [11]. Due to high requirements for quality substrate in power devices, the growth rate of crystals is slow and the preparation cost is high. For decades, people have employed many methods to improve crystal quality [12,13,14], including using seed crystals with an off-axis angle to meet the requirements of the step flow growth mode, reducing the number of defects; using different seed crystal fixation methods to reduce the stress caused by the different thermal expansion coefficients of crystal and graphite; and employing different materials and structures to optimize the internal environment of the growth chamber [15,16,17]. Steiner [18] proposed different kinds of nitrogen-doping distribution and seed-mounting strategies. Pezoldt [19] used rapid thermal processing to imprint the polytype transitions by controlling the nucleation and structural evolution during the temperature ramp-up and the steady state. Yang [20] pointed out that spiral electromagnetic coils easily generate a non-axisymmetric temperature field. In general, improving crystal growth conditions through thermal field design and optimization have always been one of the most important topics for researchers [21].

In the process of SiC crystal growth, the distribution of the temperature field plays an important role and directly affects the supersaturation of gas components at the growth interface [21,22]. Generally speaking, with the increase in crystal thickness, the source materials are gradually graphitized and recrystallized and the thermal resistance in the chamber gradually changes, which directly affects the distribution of gas phase components in the reaction chamber and has an irreversible impact on growth conditions, resulting in a low rate in the late stage of crystal growth, as well as changes in growth conditions. Therefore, the stability of growth conditions is important, but difficult to maintain, which is also the main constraint against the preparation of large-size and high-quality crystals [16,23]. Considering the high temperature state while crystal grows, the means to regulate the growth conditions are quite limited, including modifying the process parameters of temperature or power, gas pressure, the component ratio, thermal field rotation and thermal field movement [15,24]. Among these, the movement of the thermal field has the most direct effect on growth morphology. Some researchers have explored the influence of the relative height of the thermal field [20,25], but there is a lack of systematic modeling and analysis covering the crystal growth process.

In this paper, the whole growth process of 6-inch SiC crystal is modeled with the help of the Virtual Reactor simulation tool; the evolution characteristics of representative physical parameters in the growth process are analyzed, and the reasons for the change in crystal growth interface and the decline of growth rate are discussed and revealed, then compared with other experiments. During this period, we adjusted the physical parameters of the crucible and the insulation structure to match the deposition rate trend in each area to the experiment data. Since the temperature of the measuring point is kept constant, we also adjusted this based on the calculated power and the actual power. The general process can be found in previous work [26]. Then, aiming at the solution of the deterioration conditions outlined above, thermal field moving schemes with different directions and rates were designed and the distribution and evolution laws of the main physical fields under different schemes were obtained. In addition, interesting changes in crystal morphology were predicted.

## 2. Modeling and Experiment

**Figure 1:**
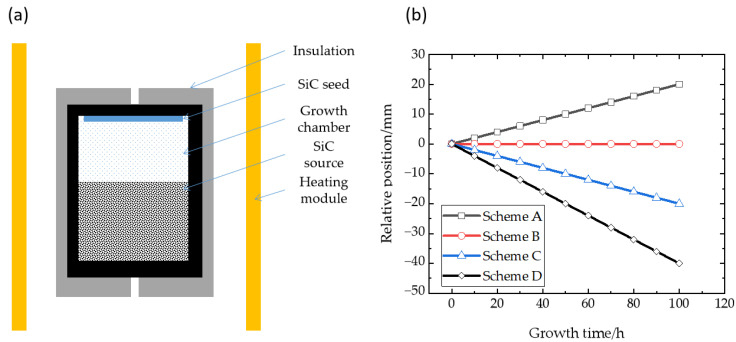
(**a**) Schematic diagram of thermal field; (**b**) Different schemes.

The crystal growth equipment is independently designed and assembled and includes several necessary subsystems to provide the basic crystal growth environment, such as a temperature measurement and control system, gas pressure control system, vacuum realization and measurement system, heating system, water cooling system, mechanical motion system, etc. The thermal field is usually located in the center of the sealed furnace body, which is supported at the appropriate position relative to the heating module by the rigid insulation material. All relevant material physical parameters can be found in previous work [26]. Of course, the appropriate thermal field position can also be obtained by the adjustment of the heating module, which is a resistance heater or induction coil. As shown in Figure 1, the relative position of the thermal field structure and the heating module is shown. The crucible body is located inside the insulation layer, the seed crystal is mounted at the top of the crucible, and the raw material is located at the bottom. An induction coil is selected as the heating module. In the initial and default positions, the difference in axial position, H, between the source material surface and the center of the heating module is +50 mm.

In order to compare the effects of different relative positions of thermal fields on the crystal growth process, the coils positions, moving in different directions and speeds, respectively, are set in different schemes, while scheme B (the original scheme) has the unchanged coils position in the whole process as a comparison. As the growth proceeds, the way that relative positions of coils have changed for each scheme is shown in Figure 1b. Among them, scheme A is set to move the coils in an upward direction, and schemes C and D are set to move the coils in a downward direction. Considering the general crystal growth rate of 100–300 μm/h, the setting of scheme C guarantees that the position of the heating zone remains approximately constant compared with the crystal growth interface, and the decline rate of scheme D is twice the expected growth rate.

Consistent with the actual crystal growth process, the main conditions for simulation calculation are shown in Table 1 as below. The temperature monitoring point is at the upper center position of the seed crystal. Under different schemes and growth times, the temperature and gas pressure are set to be constant. The output frequency of the power supply is 10 kHz and the initial power is 10 kW, which will be calculated according to the actual situation. The hot field crucible is made of R6510, while side insulation and bottom insulation are made of GFA10 and CBCF-18, respectively. The SiC raw material used is in powder form with a particle size of around 100–300μm. Thermal parameters of the main materials are also shown in the table. It should be pointed out that, with the simulation calculation, the thermal conductivity of each module is set to depend on the value of the temperature change, and does not decay or deteriorate with the actual reaction process due to the consumption. At the same time, the loss of the inner wall of the crucible is not taken into account, which usually participates in the reaction and transfer of components. The basic mathematical equation of simulation calculation is introduced and will not be repeated here [27,28,29]. A Navier–Stokes Model is employed to solve flow dynamics, and Inert Conservation is selected for the pressure evaluation model. Thermal conductivity variation is introduced to illustrate the power evolution progress. The residuals of each physical quantity in the calculation process are set based on the balance of calculation accuracy and efficiency. After meshes division of each physical domain, there are 38 blocks in the model, and 161 boundaries are formed, including 30 gas–solid interfaces, 32 solid–solid interfaces, 2 solid–powder interfaces, and 1 powder–gas interface. Delaunay and AdvancingFront are combined to generate meshes and finally form 32,000 mesh cells and 5203 boundary edges.

## 3. Results and Discussion

### 3.1. Temperature Evolution in Chamber

#### 3.1.1. Interface Temperature Distribution

**Figure 2:**
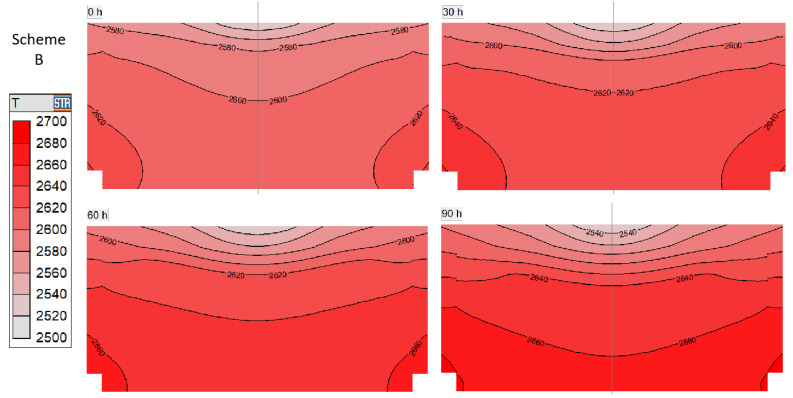
Temperature distribution of scheme B at different growth times.

During the crystal growth process, the temperature change in the growth chamber of scheme B is shown in Figure 2. As the growth reaction progresses, the isotherms gradually become denser, and the temperature distribution range in the chamber extends from 2540 to 2620 K to 2540 to 2660 K. The high temperature line gradually advances from the bottom to the top part of the chamber, and the temperature in the middle of the chamber increases at a rate of nearly 20 K per 10 h. The process of scheme B keeps a constant value at the temperature measuring point, and the relative position of the thermal field is unchanged, so the main factors affecting the thermal field are the gradually growing crystal and the gradually evolving SiC powder source. Since the center of the heating zone is located below the raw source material, the middle and lower parts of the crucible have a higher temperature. As a result, heat is transferred from the crucible wall through the growth chamber to the top low-temperature zone. Under the combined action of crystal thickening and graphitization at the edge of the powder source, the overall thermal resistance in the growth chamber increases. In order to meet the condition that the temperature at the measuring point is constant, the heating power will also increase automatically, which is conducive to increasing the chamber temperature to a certain extent during the growth process, as will be discussed later.

**Figure 3:**
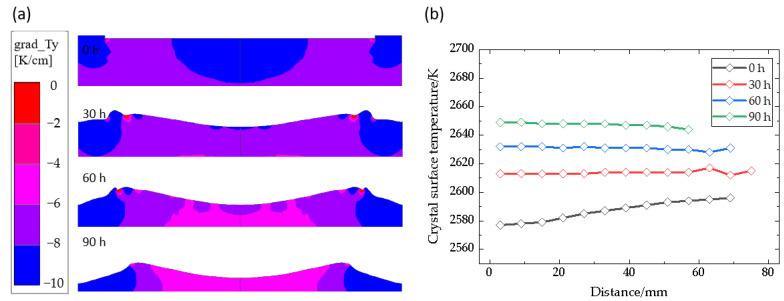
(**a**) Axial temperature gradient; (**b**) Interface temperature.

Affected by the changes mentioned above, the axial temperature gradient around the growth interface and the temperature of the crystal growth interface at different growth times are also different, as shown in Figure 3. Although the isotherms in the chamber are denser, the temperature gradient near the growth interface decreases gradually during the process, which plays a role as the driving force for crystal growth (Figure 3a). The growth interface temperature increases by approximately 20 K every 30 h, from 2590 K in the initial stage to 2650 K (Figure 3b). At the same time, the temperature along the radial direction in the early stages of growth gradually increases, with a center temperature of 2578 K and edge temperature of 2595 K. From 30 h to the final stage of growth, the temperature distribution at the center and edge of the crystal gradually draws nearer. At 90 h, the center temperature is slightly higher than the edge temperature. That is, the temperature in the center of the crystal increases greatly, while the increase in the edge region is insignificant. In addition to the law of temperature increasing at the growth interface, the length of the curve also reflects the width of the single crystal region. Growth temperature fluctuations are the main reason for the formation of SiC polytypes [30], while polycrystals are formed without seed crystals, and the polycrystal rate is twice that of the single crystal under the same conditions [31]. Therefore, there is a circle of polycrystals around the SiC single crystal. It is very important to reasonably control the growth rate of polycrystals and single crystals in SiC single crystal diameter expansion growth [32]. As the growth process progresses, the single crystal region of the crystal first expands and then shrinks, and the single crystal part is eroded by the polycrystalline part. The single crystal diameter is reduced to less than 120 mm after 90 h of growth, which greatly reduces the available diameter of the crystal, because when the crystal growth interface is too convex, it indicates that the edge temperature is too high. Therefore, the decrease of temperature gradient leads to slower growth for single crystals, and comparative advantages for polycrystalline. In general, with the progress of crystals growth, there are several problems, including temperature increasing at the growth interface, diameter decreasing of the available single crystal and temperature gradient decreasing around the interface.

#### 3.1.2. Heat Flux of Crucible

**Figure 4:**
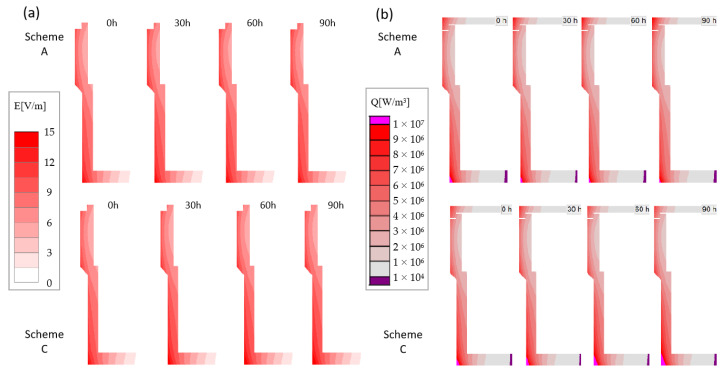
(**a**) Electric field strength; (**b**) Heat flux in crucible side during different growth time of scheme A and C.

In order to compare the heat transfer changes caused by the movement of heating models under different coils’ movement schemes, the distributions of electric field intensity and heat flux at the crucible under schemes A and C are obtained, as shown in Figure 4. The intensity of the induced electric field changes with the relative position movement of the coils. The field strength distribution at the beginning of the two schemes is the same, and the maximum field strength reaches 15 V/m. Similar to the skin effect, the field strength at the corners of the outer wall of the crucible is large, and there is still 3 V/m field strength at the center of the bottom of the crucible. In scheme A, the color of the middle position of the crucible side wall is deepened and the overall field strength of the upper part of the crucible side wall increases significantly at 30 h. This is because the height of the coils center at the initial position is below the crucible, while the outer diameter of the upper part of the crucible side wall is larger and closer to the coils. The lower part of the crucible side wall changes less because it is a little further away from the coils. It can be seen from the change in the upper part of the crucible in scheme C that the downward movement of the coil does not reflect an obviously contradictory change against that in scheme A, because during the crystal growth process, the gradual increase of the required power (see power analysis later) plays a leading role. It can be seen from the lower part of the crucible that increasing electric field strength makes the bottom color deepen. The change of heat flux on the side wall of the crucible is consistent with the change law of field strength. The heat flux is concentrated on the side wall of the crucible and changes significantly at the structural corners (Figure 4b). The heat flux distribution in the crucible changes exponentially, and the values at the bottom edge of the crucible are three orders of magnitude greater than that at the bottom center of the crucible. Since the seed crystal is located in the upper region of the crucible, the distribution of this parameter will have a significant impact on the crystal deposition conditions.

**Figure 5:**
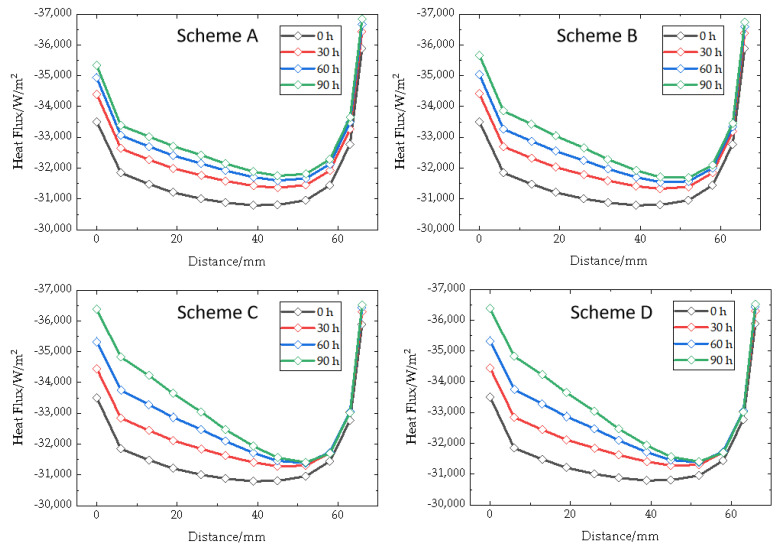
Heat flux along outer wall of crucible top part of different schemes.

Due to the significant effect of the upper part of the crucible on the growth chamber, the heat flux of the upper side wall of the crucible is compared and shown as Figure 5. This parameter refers to the heat transfer from bottom to top at the outer wall of the upper part of the crucible. A negative value indicates that the heat is going from the outside to the inside. As with the analysis mentioned above, the following three laws can be obtained. Firstly, the heat flux distribution of the upper side wall of the crucible shows the phenomenon of large values around the edge and small values in the middle. Near the top part of the crucible, the heat flux changes violently. Second, heat flux has the minimum value at the position 15 mm away from the top of the crucible, but it does not change significantly with the movement of the coils. Third, with the growth process, the heat flux gradually increases, but its increasing range decreases. The added value of heat flux decreases when the coils move upward, and when coils move downward, the increased value of heat flux gradually increases. The area with the maximum variation is between positions 0 and 20 mm, which are near to the middle of the crucible and the middle of the coils. This shows that the position change of the heating area within the crucible is not obvious due to the shift in the coils position.

#### 3.1.3. Temperature Difference between Powder and Seed Crystal

**Figure 6:**
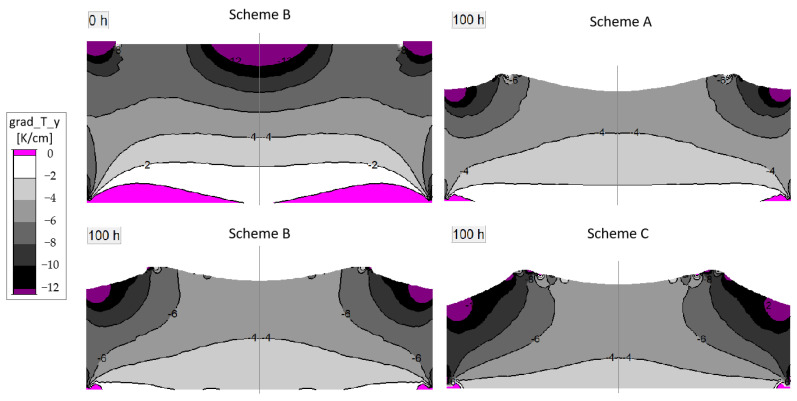
Temperature gradient in growth cell of different schemes.

The internal conditions of the crucible are worthy of much more attention. Figure 6 shows the axial temperature gradient distribution in the growth chamber under schemes A, B and C at the initial time and 100 h. Regarding the growth process, the gradient at the center of the crystal decreases significantly, and its features shift from large in the middle and small at the edge to the contrary. This indicates that the deposition rate at different positions changes rapidly during the growth process. Compared with scheme A, the edge region of scheme C maintains a higher temperature gradient when the coil moves downwards. This indicates that this condition is conducive to maintaining a higher deposition rate. Meanwhile, the parameter value of the temperature gradient in the edge region of scheme C fluctuates greatly, which may be detrimental to the crystal quality.

**Figure 7:**
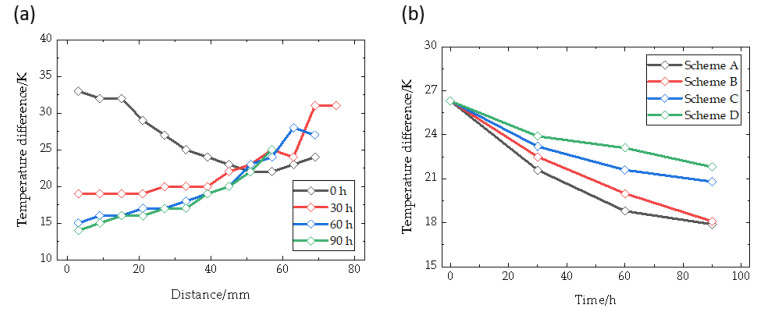
Temperature difference between powder and seed crystal. (**a**) Scheme B; (**b**) Average values of all schemes along growth time.

In order to further compare the gradient differences among different schemes, Figure 7a introduces the temperature disparity between the raw source material surface and the seed crystal surface of scheme B along the center to the edge. It is only at the initial time of growth that the central position of the crystal maintains a large temperature gradient, and this parameter rapidly decreases from 33 K to below 20 K at 30 h, which is in good agreement with the convex shape of the central position during crystal growth. The deterioration of gradient is the key factor affecting the consistency of crystal deposition rates. Figure 7b shows the average value of temperature differences shifted among raw material seed in different schemes. Compared with the original scheme B, the coils moving downwards alleviates the problem of gradient reduction to a certain extent. Furthermore, the faster the movement, the slower the gradient attenuation. When the coils’ positions move downwards at a rate of 400 μm/h, the average temperature gradient is 20% higher in 90 h, which shows a significant impact. Of course, the trend is reversed when the coil moves upwards. This indicates that the coil movement provides the possibility of alleviating the gradient degradation.

### 3.2. Source Material Evolution

**Figure 8:**
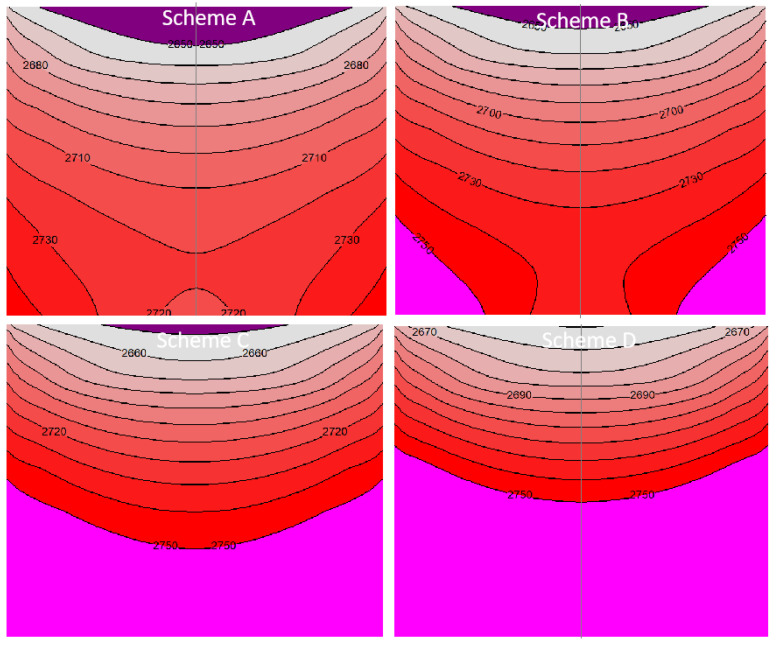
Temperature distributions of powder of different schemes.

**Figure 9:**
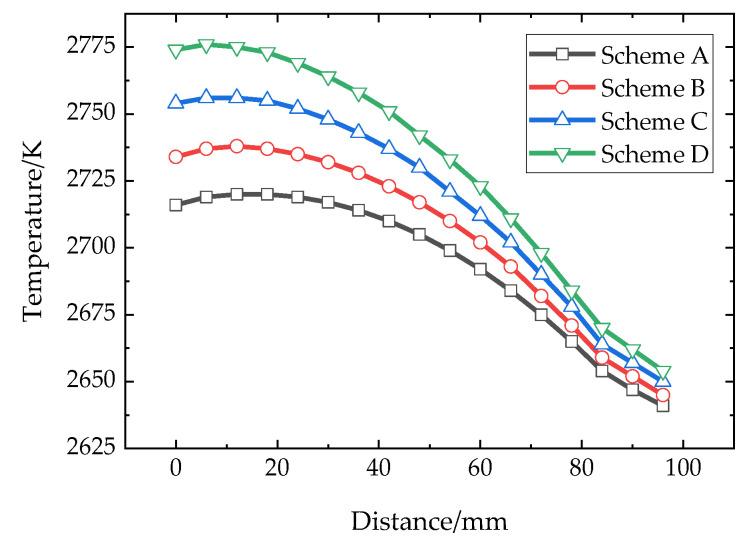
Temperature distributions along axial directions of different schemes.

SiC raw materials provide the necessary vapor components for the crystal growth, and their evolution is very important. The temperature distribution inside the raw material at 50 h under different schemes is shown in Figure 8. Compared with the original scheme B, the raw material temperature reduces about 15 K when the coil moves upwards, with the overall temperature between 2640 and 2740 K. Under the conditions of scheme C and scheme D, the temperature at the top and bottom of the raw material increases significantly, and the isotherm of 2750 K even moves upward to the middle of the raw material. Meanwhile, the isotherm becomes denser and smoother. The reason needs to be discussed in combination with the evolution features of raw materials. During the growth processes, raw materials at the edge are decomposed and gasified because they are close to the crucible wall, with the highest temperature, thus reducing the thermal resistance at the edge and increasing the temperature of the core area of raw materials. Figure 9 shows the temperature distribution of the symmetry axis of raw materials from bottom to top when the growth process is at 50 h. From schemes A to D, the place where the temperature changes most is at the bottom of raw materials, and the temperature disparity between schemes reaches about 20 K, while the temperature change near the top of raw materials is smaller with the total temperature difference of 20 K. It indicates that the temperature change of the raw material surface is less obvious than that of bottom part.

**Figure 10:**
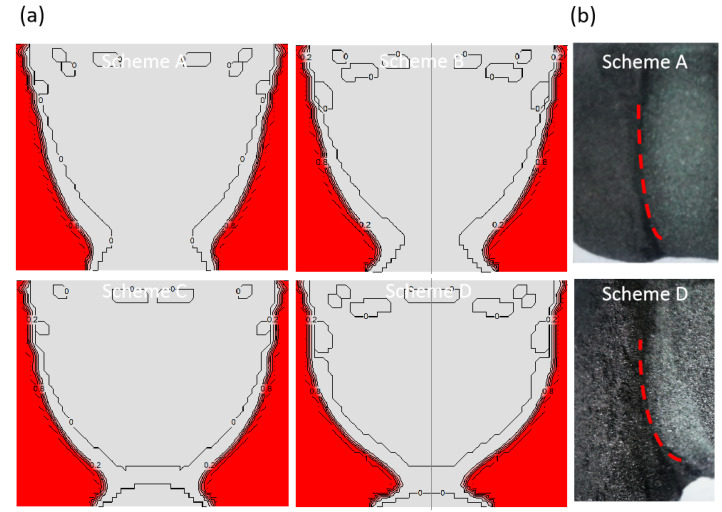
(**a**) Source graphitization degree; (**b**) Source in experiment.

**Figure 11:**
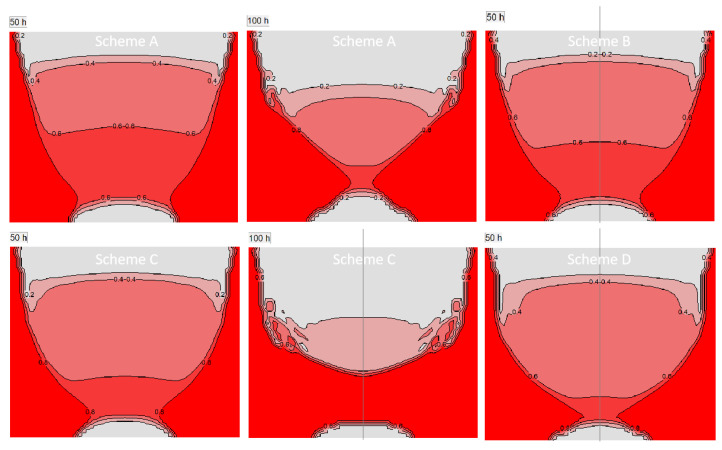
Porosity of powder source in different schemes and growth time.

The heat flux change brings the shift in the internal temperature distribution of raw materials, while the internal temperature of raw materials changing results in the development of the graphitization and recrystallization of raw materials. The change of internal thermal resistance of raw materials then affects the later overall heat transferring to the growth chamber. Figure 10a shows the predicted graphitization degree within the raw materials under the four schemes at 50 h, which is similar to the temperature distribution. The crimson region indicates that this part is completely graphitized. The raw materials at the side wall of the crucible are graphitized rapidly, and the graphitization degree gradually increases in the downwards direction, but there is a turning point near the bottom of the crucible, which shows that the temperature near the bottom of the crucible is lower than that of the bottom side wall of the crucible. It is because the surface and bottom of the raw materials have relatively low temperatures, and the concentration of gas components is higher, that recrystallization occurs within these areas. In contrast, the actual experimental raw material change matches well with the simulation. After the severe graphitized raw material is removed, graphite edge contours are shown as red dotted lines (Figure 10b). When the coils move downward, the graphitization of raw materials near the bottom becomes more obvious. Figure 11 shows the porosity changes of these various schemes. The porosity of the raw material is positively related to its graphitization degree, while there are obvious areas with lower values at the top and bottom of the raw material, which means that the raw material in this area has recrystallized because of the presence of proper temperature gradients. The recrystallization of the upper part of the raw materials makes the thermal resistance of the top of the raw material change less during growth, which is conducive to the formation of the stable temperature conditions within the chamber. Meanwhile, due to the progress of recrystallization and the reduction of porosity, the formation rate of feed gas components will also be reduced, which will interfere with the maintenance of crystal growth supersaturation. At 100 h, the comparison between schemes A and C is obvious. The upper part state is similar, but the degrees of graphitization and recrystallization under scheme C are higher than that under scheme A, which indicates that more raw materials enter the reaction gas phase components.

### 3.3. Growth Rate and Morphology

**Figure 12:**
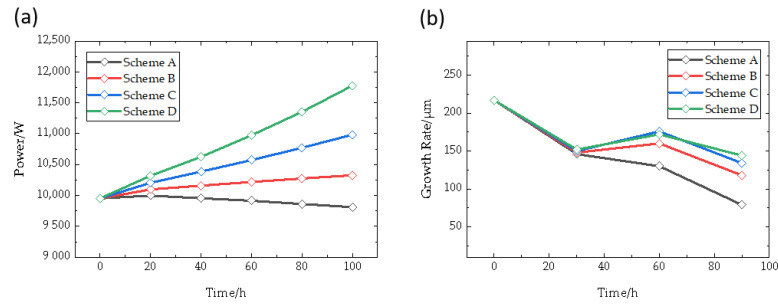
(**a**) power required during different time; (**b**) Growth rate of different schemes.

After simulating the deposition process, the parameters of growth rate and required power under different schemes are obtained, as shown in Figure 12. Under schemes A to D, as the heating area changing is a result of the movement of the coils, the required power changes slowly to keep a constant temperature. Among them, scheme B slowly increases by about 400 W during the whole process. As a comparison, scheme D increases by about 1750 W and scheme A decreases by about 200 W. For scheme A, the power decreases slowly from peak values at around 20 h. Combined with the analysis mentioned above, during the growth process, the change of thermal resistance in the chamber caused by recrystallized raw materials and thickened crystals in this stage is gradually reduced. Meanwhile, the heat transfer efficiency is improved, resulting in a small decrease in the required power. Considering that the power change in Scheme B is only influenced by crystal growth and raw material graphitization, it can be concluded that the overall thermal resistance inside the crucible increases during crystal growth. However, as the coils’ movement has a greater impact on the required power under the four schemes, the average growth rates of growth interfaces at different growth stages are 140, 152, 165 and 172 μm/h, respectively. First of all, although the growth rate gradually decreases with the continuation of the growth process, schemes C and D have higher growth rates than scheme B. Meanwhile, the crystal growth rate of scheme A decreases significantly, reaching more than 60%. This verifies the inference that the coils could be moved downward to improve the deterioration of the temperature gradient. Compared with scheme C, the effect of scheme D is not very significant. By moving the coils downward near the crystal growth rate, a more ideal deposition rate is obtained at a constant temperature. In addition, the crystal growth rate at 60 h is higher than that at 40 h, which also verifies several facts from the side. One thing is the physical quantities in the crystal growth process are dynamic changes, which are coupled with each other; the other is that the deposition state is determined by the joint action of multiple factors.

**Figure 13:**
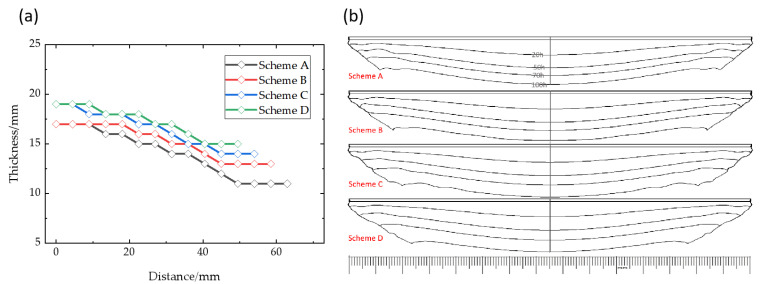
(**a**) Crystal thickness of different diameters; (**b**) Crystal profile predicted of different schemes.

The crystal thickness and surface profile under different schemes are shown in Figure 13, in which the distance 0 mm refers to the center of seed crystal. The driving force of crystal growth is the gas component supersaturation, so the growth rate is closely related to temperature conditions. In schemes A to D, the crystal thickness gradually increases, and the thickest parts of schemes C and D are about 2 mm thicker than schemes A and B. The convexity of each scheme is 6 mm, 4 mm, 5 mm and 4 mm, respectively. Scheme A has the steepest surface change, while the convexity of schemes C, D and scheme B is similar. However, in terms of single crystal diameter, the crystal diameter of each scheme is reduced by 5 mm, and scheme A, with the slowest growth rate, has a larger single crystal diameter. As Figure 13b shows, the crystal growth thicknesses in different schemes are basically the similar from the beginning to 20 h growth time, and are not affected by the movement of the moving coils. During the growth process, the thickness increase of scheme A is always the least at each growth stage, while the growth rate of schemes C and D is faster, and the growth rate of the crystal edge is faster than that of the central region. At the same time, the angle of the side edge of the crystal profile is about 40°, 35°, 33°, 30°, which means that the original axial growth conditions cannot be maintained. It can be seen from the crystal edge contour that the diameter of the crystal often decreases sharply when the coils move down a node. This is because, under such conditions, the downward movement of the coils increases the growth rate of the crystal, but the deposition rate of the polycrystalline is even faster. Therefore, the diameter of the single crystal region is compressed, which is consistent with the conclusion that it is conducive to crystal growth under the condition of lower growth rates.

## 4. Conclusions

The entire growth process of a 6-inch SiC is modeled with the help of the VR software, as the evolutionary features of representative physical parameters in the growth process are analyzed. Aiming at the crystal growth interface and growth rate improvement, the effects of different movement schemes of the thermal field on the distribution are analyzed, while the evolution of the physical fields and growth processes under the conditions of constant temperature measurement point are systematically compared for the first time. Results show that, during crystal growth, the attenuation of raw materials has a great influence on crystal growth conditions, which is the primary reason for crystal surface temperature increasing and temperature gradient decreasing. Meanwhile, the upward movement of the thermal field is conducive to reducing the deterioration of the temperature gradient and maintaining the growth rate at a certain level, while the latter is increased from 152 μm/h to 165 μm/h, with a range of 8.5%. When the movement speed is further accelerated, the rate rises to 172 μm/h. The downward movement of the thermal field is conducive to the growth of single crystal diameter expansion under experimental conditions. This is because the effects of thermal field movement on the single crystal rate are less obvious than that on the edge polycrystals. The downward movement of the thermal field helps to expend the diameter of the single crystal region. Compared with the fixed position scheme, the diameter of the growth surface increases by 5 mm after 100 h of growth and its convexity is improved. This work provides a useful reference for adjusting the position of the thermal field and improving the crystal edge and deposition speed.

## Figures and Tables

**Table 1:** Main calculation conditions.

Parameter		Values
Growth conditions	Monitor point temperature (°C)	2250
	Frequency (Hz)	10,000
	Initial power (W)	10,000
	Pressure (Pa)	2000
Electric conductivity (Ω^−1^·m^−1^)	Inductor	5.5 × 10^7^
	Crucible	1.065 × 10^5^ (2250 °C)
Thermal conductivity (W·K^−1^·m^−1^) (2250 °C)	4H-SiC	18.4
	Crucible	29.5
	Side insulation layer	1.13
	Bottom insulation layer	1.37
Residual	RF heating	1 × 10^−7^
	Temperature	1 × 10^−6^
	Species	1 × 10^−5^
	Velocity	1 × 10^−4^
	Pressure	1 × 10^−4^
	Growth rate	1 × 10^−4^

## Data Availability

Data are contained within the article.
